# Heterogeneity of the Impact of the Social Old-Age Insurance and the Medical Insurance on the Mortality Risk of the Elderly

**DOI:** 10.3389/fpubh.2022.807384

**Published:** 2022-03-04

**Authors:** Wangchun Wu, Chunhua Li, Bin Gao

**Affiliations:** ^1^School of Finance, Nankai University, Tianjin, China; ^2^School of Economics, Guangxi University for Nationalities, Nanning, China

**Keywords:** heterogeneity, old-age insurance, medical insurance, mortality risk, frailty Cox model

## Abstract

Based on the frailty Cox model, this paper analyzes CLHLS data from 2008 to 2017/2018 to examine the impact of the social old-age insurance and the medical insurance on the mortality risk of the elderly based on the age structures, urban/rural areas and regions. The results reveal the heterogeneous impact as follows. In terms of the age structures, the social old-age insurance significantly reduces the mortality risk of the elderly aged below 80, but has no significant impact on the elderly aged 80 and above, whereas the medical insurance significantly reduces the mortality risk of the elderly aged 80 and above, but has no significant impact on the elderly aged below 80. In urban/rural areas and different regions, the social old-age insurance has no significant impact on the mortality risk of the elderly, whereas the social medical insurance significantly increases the mortality risk of the elderly in urban areas and the East, and reduces that of the elderly in rural areas and the Middle and the West. When implementing the insurances, China should pay attention to the different attributes of the elderly to guarantee the service quality, including the age structures, urban/rural areas and regions. A full consideration should be given to the allocation of investment and social security resources, so as to address the issue of the mismatch between the supply and demand of medical resources, and finally achieve the success of healthy aging and health equity.

## Introduction

*The 14th Five-Year Plan* in China proposes that we should develop a fair, unified, and sustainable multi-tiered social security system that covers the entire population in both urban and rural areas. Social insurance is an important part of the social security system, in which the social old-age insurance and the social medical insurance are the core content. At present, China's “Basic Old-age Insurance for Urban and Rural Residents” is the joint name of “The New Rural Old-age Insurance (NCMS)” and “The Old-age Insurance for Urban Residents.” The pilot projects were launched in 2009 and 2011, respectively. In 2014, they were jointly implemented (1). In terms of the Basic Medical Insurance, some regions are in the leading position of the insurance integration, especially in developed regions. For example, Dongguan City in Guangdong Province was the first to realize the integration in 2008. In January 2016, the State Council issued *The Opinions on Integrating the Basic Medical Insurance System for Urban and Rural Residents*, requiring the whole nation to promote the integration of The Medical Insurance System for Urban Residents and The New Rural Cooperative Medical System, and gradually establish a unified medical insurance system for urban and rural residents. So far, almost all regions in China have completed this task (2).

After years of concerted efforts by the Party and the responsible departments, a social security system covering China's whole population is established. The Basic Old-age Insurance and The Medical Insurance for Urban and Rural Residents are continuously improving the social equity and producing extensive social benefits in China. However, the primary purpose of the integration is to realize social equity by eliminating the urban-rural dual structure and balancing the regional development. Has the expected purpose been achieved now? Since 1999, China has entered the aging society, and the population of the elderly is rapidly growing. By the end of 2020, the elderly population aged 65 and above accounts for 13.5% of the total population, meaning that China will enter the super aging society by 2030 (3, 4). With the deepening and acceleration of China's aging problem, the proportion of the elderly aged 80 and above is growing fast. What health benefits do these two social insurances bring to the elderly under different age structures? What is the impact on the mortality risk of the elderly? What is the functional scale, direction and mechanism? This paper is looking for the answers.

The rest of the paper is organized as follows. Section Literature Review reviews and concludes the findings of previous studies and literature. Section Research Methodology and Data Sources introduces the data sources and research methodology. Section Regression Result is the empirical results and Section Discussion and Conclusion is the conclusion and discussion.

## Literature Review

Previous researches on the impact of the social old-age insurance and the social medical insurance on the health of the elderly mainly focus on three aspects.

First, the positive impact on the health of the elderly. Li and Yang use the urban household survey data from CHIP 2002 and 2007 to analyze the impact of pension on the health of the urban elderly. The results show that there is a positive correlation between the pension level and the self-rated health. Pension is more and more important in ensuring the health of those living alone (5). Huang and Gan use data from CLHLS 2002-2005, and conduct a research with the two-part model, the extended sample selection model and the Cox proportional risk model, finding that the mortality risk of the urban elderly covered by medical insurance is lower than that of the uninsured ones (6). In studying rural residents, Cheng and Zhang use the two-part model, the Heckman selection model and the Probit model to estimate the impact of NCMS on the actual medical expenditure of the rural elderly, finding that NCMS significantly improves the health status of the participating elderly (7). Using data from CLHLS 2008 and 2011, with the fixed effect panel model and PSMDD, Chen and Zeng find that NCMS can improve the welfare of the rural elderly (8). Sebastian et al. use the quasi experimental design method to study the non-contributory pension in Mexico, finding that the non-contributory pension reduces the depression value of the elderly, improves their mental health significantly, and improves the well-being of the poor elderly (9).

Second, the insignificant impact on the health of the elderly. Different from the above researches, Finkelstein and McKnight find that the universal medical insurance system established in 1965 in the United States has no significant impact on the mortality of the elderly in the first 10 years after its implementation (10). Polsky et al. analyze the data from 1992 to 2006 of the United States with the quasi experimental method, and find that there is little difference in health improvement between the elderly with medical insurance and the elderly without (11). Based on the two-stage panel data of CHARLS, Chinese scholar Xie adopts the difference-discontinuity method and concludes that NCMS has no influence on the depression index which reflects the mental health (12).

Third, the heterogeneity of the impact on the health of the elderly. To answer the question of whether the insurances affect the health of the elderly, some scholars analyze the problem from the perspective of heterogeneity. Huang and Wu use data from CLHLS 2002-2005 with the discrete-time analysis method, and find that the mortality rate of the urban elderly aged 60 without the medical insurance is higher than that of those with the insurance, but is lower after the age of 96 (13). Using data from CLHLS 2005-2011, Wang uses the propensity score matching method and the multi-stage difference in differences model to find that NCMS truly improves the health of the elderly, but decreases their self-rated health (14). Based on the data from CLHLS 2008-2011, Xu and Liu use PSMDD method and the intermediary effect model to find that NCMS significantly improves the physical health of the elderly, but worsens their mental health (15). With data from CLHLS 2014, Yang and Xiao use the structural equation model to estimate that pension significantly improves the mental and physical health of the elderly, whereas the expenditure of the medical insurance plays a negative role (16). Based on the data from CLHLS 2005-2014, Guo and Gu find that the relations between the medical service accessibility and the health of the elderly vary with gender, age and urban/rural areas (17). Based on the CGSS data in 2013, Liu and Wang use the ordered logistic regression model to find that both the social old-age insurance and the medical insurance significantly improve the physical, mental and self-rated health of the urban elderly, yet only the social medical insurance significantly improves the physical health of the rural elderly (18). With the CHARLS data in 2015, Zhou et al. use the structural equation model to draw a conclusion that the basic medical insurance significantly improves the health of the urban elderly, but the impact of NCMS is not so significant (19).

From the above discussion, we see that researches on the impact of the social old-age insurance and the social medical insurance on the health of the elderly has developed from an earlier stage—whether they have significant impact on the overall population—to a profound and objective stage with multi-dimensional perspectives. Scholars study the problem from one single perspective of either focusing on the social old-age insurance or the social medical insurance, to a comprehensive comparative analysis of both. In terms of health and welfare of the elderly, researchers discuss on the physical health, mental health, self-rated health and mortality risk of the elderly. Research data come from continuously updated sources like CLHLS, CHARLS, CGSS, and CHIP, and the research methods are increasingly diversified.

The research contents, research perspectives, research methods and research conclusions from the above provide a broad view and lay a solid foundation for this study. The research gap entails: (1) Most studies focus on the health of the surviving elderly, including physical health, mental health and self-rated health, but rarely extend to the end of life—mortality. In terms of the variables on the health of the elderly, with the help of advanced medical technology, recovery aids and intelligent communication tools, the elderly with poor mobility can overcome the short board of common indicators such as Activities of Daily Living (ADL) and Instrumental Activities of Daily Living (IADL) (20, 21), thus blurring the boundary of ADL or IADL measurement indicators. Some indicators reflecting mental health are mainly based on researchers' understanding and judgment, instead of the diagnostic results of professional psychologists, leading to the subjectivity of the variables to a certain extent. If we analyze the ultimate state of health—mortality, we can effectively avoid the measurement problem of the indicators to some extent, and can also measure the results of the development and change of physical health, mental health, and self-rated health. (2) In recent years, many researchers have noticed the heterogeneity of the social old-age insurance and the social medical insurance on the health benefits of the elderly, but they mainly focus on the differences between urban/rural areas and health types, instead of different regions and age structures. The economic development and the resources of insurance vary in the East, Middle, and West of China (22), thus the impact on the mortality risk of the elderly is certainly different. For the elderly aged 80 and above, due to the declination of physiological ability, they have a greater demand for the medical insurance and it may have a greater impact on their health than the social old-age insurance. (3) The data in the previous studies are relatively outdated. The latest research uses data from the year of 2015. It does not reflect the current changes of the access to the social old-age resources and the public medical resources, neither the impact of the current changes on the health of the elderly.

This paper uses the latest data to study the overall impact of the social old-age insurance and the medical insurance on the mortality risk of the elderly, and explore the heterogeneity and its causes under the samples of age structures, urban/rural areas and regions. Suggestions are provided in the last part to help improve the social insurance system and targeted governance, and build a healthy aging society.

## Research Methodology and Data Sources

### Data Sources

This study uses data from the four follow-up surveys in China Elderly Health Influencing Factors Tracking Survey (CLHLS) from 2008 to 2017/2018 conducted by PKU Center for Healthy Aging and Development, which covers 23 provinces, cities and autonomous regions in China. After the baseline survey in 1998, the follow-up surveys were conducted in 2000, 2002, 2005, 2008-2009, 2011-2012, 2014, and 2017-2018, respectively. CLHLS is an internationally recognized project, which covers the largest number of samples compared to all the other health surveys on the elderly in the whole world. It contains abundant data and has huge research potentials. Till October 15, 2021, more than 10,327 scholars from home and abroad have published papers based on its survey data and the data quality is widely recognized^1^. As most of the elderly in the early surveys have passed away, this study analyzes the follow-up survey data of the last decade.

Considering the poor data quality of the elderly aged above 105, data of the elderly aged 65-105 were selected. 12,564 sample were finally chosen after eliminating the missing value and unsuitable answers. Among the 12,564 samples, 5,043 samples passed away before the survey in 2011; 2,337 samples passed away before the survey in 2014; 1,399 samples passed away before the survey in 2017/2018. During the observation period, 8,779 samples passed away, 1,626 lost contact, and 2,159 are alive.

### Research Methodology

The Cox proportional risk model is widely used in the study of mortality risk. We first define the cumulative mortality rate, which represents the probability of variable X's observation subject when the survival time T is not greater than time t:


(1) $$\begin{array}{l} \text{D}\left(t,X\right)=P\left(T\leq t,X\right) \end{array}$$

Here we study two core independent variables—the social old-age insurance and the social medical insurance, as well as the impact of the control variable set X on the mortality risk of the elderly, that is, the relations between X and D (T, x). However, as many factors influence on D (T, x) and the time variable t is difficult to meet the requirements of normal distribution and homovariance, the traditional method of direct regression estimation of X and D (T, x) is unsuitable here. In equation (2) and equation (3), we define the instantaneous mortality rate of variable set X's observation subject at time t as *f***(t, X)** and the conditional instantaneous mortality rate at time t when the survival time reaches t as μ**(t, X)**. μ**(t, X)** is used as the dependent variable in the Cox model, as shown in equation (4).


(2) $$\begin{array}{lll} f\left(t,X\right) & = & \underset{\Delta t\to 0}{\lim}\frac{P\left(t<T\leq +\Delta t,X\right)}{\Delta t} \end{array}$$

(3) $$\begin{array}{lll} \mu \left(t,X\right) & = & \underset{\Delta t\to 0}{\lim}\frac{P\left(t<T\leq t+\Delta t|T\geq t,X\right)}{\Delta t}=\frac{ft,X}{1-D\left(t,X\right)} \end{array}$$

(4) $$\begin{array}{lll} \mu \left(t,X\right) & = & \mu_{0}\left(t\right)\exp\left(\beta_{1}X_{1}+\beta_{2}X_{2}+...+\beta_{n}X_{n}\right)\\ = & \mu_{0}\left(t\right)e^{X\beta } \end{array}$$

When equation (4) satisfies the proportional risk assumption **μ(*t, X*)/μ_0_(*t*)** which does not change as time t changes, it can be transformed into:


(5) $$\begin{array}{l} \ln\left(\frac{\mu \left(t,X\right)}{\mu_{0}\left(t\right)}\right)=\beta_{1}X_{1}+\beta_{2}X_{2}+...+\beta_{\text{n}}X_{n} \end{array}$$

Equation (5) is a log-linear model. Assumptions are not needed for the baseline risk μ_0_**(t)**, as it obtains effective estimation even when μ_0_**(t)** is unknown. However, model (4) and model (5) require homogeneous research subjects. Different people have different situation in mortality, thus some observation subjects are frailer than the others (23), and there exists certain heterogeneity—Vaupel calls it as frailty (24). Frailty is an “unobserved heterogeneity,” and a common practice is to put it into the error term in the model. However, if the unobserved heterogeneity is related to some explanatory variables, the estimated coefficients may be biased and the problem of ignoring variable bias occurs. Though some models have been developed in the academic world, such as the instrumental variable estimation, the fixed effect model, the random effect model and the finite mixture model, the assumptions of different models on the distribution of unobserved heterogeneity and its relations with explanatory variables could be different. Due to various factors, the unobserved heterogeneity is difficult to be included in the model measurement in practice. The concept of frailty provides a simple method of measurement, which is used in the analysis of survival data in the frailty Cox model. Under the circumstance of heterogeneity or multiple different baseline risks, equation (4) can be improved as:


(6) $$\begin{array}{l} \mu \left(t,X,\lambda_{i}\right)=\mu_{0}\left(t\right)ex\text{p}\left(\beta_{1}X_{1}+\beta_{2}X_{2}+...+\beta_{\text{n}}X_{n}+\lambda_{i}\right) \end{array}$$

Or expressed as:


(7) $$\begin{array}{l} \mu \left(t,X,Z_{i}\right)=Z_{i}\mu_{0}\left(t\right)ex\text{p}\left(\beta_{1}X_{1}+\beta_{2}X_{2}+...+\beta_{\text{n}}X_{n}\right) \end{array}$$

Of which:


(8) $$\begin{array}{l} \lambda_{i}=\ln\left(Z_{i}\right) \end{array}$$

The variable *Z*_*i*_ is the variable, or the frailty factor as we say, to measure the heterogeneity. Equations (6) and (7) are the frailty Cox model, which can be effectively estimated by the following log-linear model.


(9) $$\begin{array}{l} \ln\left(\frac{\mu_{i}\left(t,X\right)}{\mu_{0}\left(t\right)}\right)=\beta_{1}X_{1}+\beta_{2}X_{2}+...+\beta_{\text{n}}X_{n}+\lambda_{i} \end{array}$$

The ordinary Cox proportional risk model does not consider the unobservable heterogeneity of the elderly, which overestimates or underestimates the real situation that influences the dependent variables, and the significance may change or even sometimes leads to completely opposite direction (25). Hence, to correct the deviation, it is necessary to include frailty factors in the Cox proportional risk model to form a frailty Cox model (26–28).

For equation (9), referring to the methods of Prentice and Gloeckler (29) and Meyer (30), we use the maximum likelihood estimation to acquire the statistical results from the Cox proportional risk model (“Cox model” for brevity), and the Cox proportional risk model with frailty factors (“frailty Cox model” for brevity).

### The Settings of Variables

This study runs model 1-4 with the total samples and model 5-26 with the sub samples, among which model 5-12 under the samples of different age structures, model 13-20 under the samples of urban/rural areas, and model 21-26 under the samples of different regions. During the process, we run the models with and without other control variables. Results are listed in the following chapter.

### The Measurement of Variables

In this study, the dependent variable is the mortality risk of the elderly from 2008 (base year) to 2017/2018 (end year), which has been obtained from the variable of survival time. For the elderly who are still alive in 2017/2018, the years of survival is calculated by subtracting the base year from the year of survey (2017/2018). For the elderly who have passed away during the observation period, the years of survival is calculated by subtracting the base year from the year of death. For the elderly who lost contact, the years of survival is calculated by subtracting the base year from the year of the last survey.

Two core independent variables are: the social old-age insurance and the social medical insurance which are formed in the question of the questionnaire: “Do you have the following social insurance or commercial insurance?” We regard those who answered “Yes” in the choice of “Public Old-age Insurance” as having the social old-age insurance, and those who answered “Yes” in the choice of “The Medical Insurance for Urban Employees,” “The Cooperative Medical Insurance for Urban Residents” or “The New Rural Cooperative Medical Insurance (NCMS)” as having the medical insurance. These two core independent variables are binary dummy variables, in which “Yes” is assigned as 1 and “No” as 0.

Considering the factors of residence, age, gender, marriage status, education level, residential pattern, source of income, health status, and lifestyle (31–33), we adopt the indicators that reflect these elements as the control variables. They are obtained from the indices including the old people's current residence, exact age, gender, marital status, years of schooling, living conditions with the offspring, main economic sources of life, the ability of the elderly in six daily activities (bathing, dressing, going to the toilet, indoor activities, defecation, and eating), whether they often smoke, drink or do exercise, etc.. Variables except for the variables of region, gender and education are regarded as time-varying variables. The changes of these variables have different impact on the dependent variables in the four periods of the survey. The settings of each variable and its distribution in the base period are shown in Table 1.

**Table 1:** The settings of variables and the distribution in the base period.

**Variable**	**Mean value**	**Standard deviation**	**Minimum value**	**Maximum value**
The social old-age insurance (yes = 1)	0.22	0.42	0	1
The social medical insurance (yes = 1)	0.72	0.45	0	1
Age group (aged 80 and above = 1)	0.73	0.44	0	1
Residential area (rural = 1)	0.36	0.48	0	1
Region (east = 0, middle = 1, west = 2, northeast = 3)	1.23	0.98	0	3
Gender (female = 1)	0.57	0.50	0	1
Marital status (have a spouse = 1)	0.33	0.47	0	1
Education level (educated = 1)	0.37	0.48	0	1
Residential pattern (live with the offspring = 1)	0.54	0.50	0	1
Source of income (from the offspring = 1)	0.66	0.47	0	1
ADL (need aids = 1)	0.21	0.40	0	1
Self-rated health (good = 1)	0.44	0.50	0	1
Smoke (yes = 1)	0.18	0.38	0	1
Drink alcohol (yes = 1)	0.18	0.38	0	1
Physical exercise (yes = 1)	0.27	0.45	0	1

*The total number of samples is 12,564*.

Table 1 shows that in the base year of 2008, 22% of the samples have the social old-age insurance, and 72% have the social medical insurance. Most of the samples have the following attributes: aged 80 and above, live in rural areas in the Middle and West, women, widow, have not received education, live with their offspring, and the main source of income is provided by their offspring; no smoking and drinking, and exercise less. All of the above are the current feature of the elderly in China. It should be noted that most samples have good Activities of Daily Living (ADL), and only 21% need aids. The possible reason is that to some extent, advanced medical auxiliary equipment reduces the elderly's dependence on others. However, only 44% of the elderly mark “good” on their self-rated health, meaning that the real health status of the elderly is not so good as expected.

Apart from that, according to the statistics, we learn that in the four surveys, the proportion of the social old-age insurance is 22, 38, 45, and 53%, respectively, whereas the proportion of the medical insurance is 72, 74, 81, and 85%, respectively.

## Regression Result

In this part, considering the literature review and the heterogeneity of different age structures and different regions, we discuss about the impact of the social old-age insurance and the social medical insurance on the mortality risk of the elderly under the total samples, samples of different age structures, samples of urban/rural areas, and samples of different regions.

### The Overall Samples

In terms of the overall samples, we analyze the impact of the social old-age insurance and the social medical insurance on the mortality risk of the elderly with and without control variables, respectively. The statistical results of the Cox proportional risk model and the frailty Cox model are listed in Table 2 for a comparison.

**Table 2:** The regression results of the Cox model and the frailty Cox model under the overall samples.

**Variable**	**Cox model**		**Frailty Cox model**	
	**Model 1**	**Model 2**	**Model 3**	**Model 4**
The social old-age insurance	−1.41***	0.05	0.85**	0.25
The social medical insurance	−1.57***	−1.14***	−3.44***	−0.34
Age group		0.19***		0.72***
Residential area		−0.81***		−1.15***
Gender		−1.12***		−3.22***
Marital status		−0.35***		−0.32
Education level		−1.20***		−3.11***
Residential pattern		−0.81***		−1.22***
Source of income		−0.87***		−1.19***
ADL		0.39***		1.56***
Self-rated health		−0.99***		−0.84***
Smoke		0.18***		0.38
Drink alcohol		0.25***		−0.36
Physical exercise		0.26***		0.34
Frailty factors		-	5.06***	4.59***

(1) For brevity, the standard error is not listed in the table; (2) ***, **, and * are significant at the levels of 1, 5, and 10%, respectively. The same below.

Table 2 reveals that before adding control variables, in the Cox model (Model 1) and the frailty Cox model (Model 3), both the social old-age insurance and the social medical insurance significantly have impact on the mortality risk of the elderly at the level of 5%. The social old-age insurance reduces the mortality risk of the elderly in the Cox model, but it increases the risk in the frailty Cox model.

When other control variables are added, in the Cox model (Model 2) and the frailty Cox model (Model 4), the social old-age insurance increases the mortality risk of the elderly, but is not significant at the level of 10%. The social medical insurance reduces the mortality risk of the elderly, with a significant impact at the level of 1% in Cox model and an insignificant impact at the level of 10% in the frailty Cox model. Moreover, comparing Model 2 and Model 4, we find that the independent variables have a greater impact on the dependent variables in the Cox model. For example, the variables of marital status, smoke, drink alcohol and physical exercise in Model 2 are significant at the level of 1%, but insignificant at the level of 10% in the frailty Cox model. In addition, Model 4 shows that the added frailty factors have an enormous impact on the mortality risk of the elderly. We also see that if the frailty factors are not considered, the effect of the social old-age insurance on the mortality risk of the elderly is underestimated by 23% (= exp^0.05^-exp^0.25^ = 105-128%), whereas the effect of the social medical insurance is underestimated by 39% (= exp^−1.14^-exp^−0.34^ = 32-71%).

From the test results, we know that the *p*-values of the LR tests are all 0, indicating that Model 3 is better than Model 1 and Model 4 is better than Model 2. Hence, this paper adopts Model 4 as the final interpretation model under the total samples. In Model 4, when we control other factors, the social old-age insurance increases the mortality risk of the elderly, but the social medical insurance reduces it, and neither of them is significant at the level of 10%. In terms of control variables, the urban elderly have a lower mortality risk than the rural elderly; the elderly aged 80 and above have a higher mortality risk than the elderly aged below 80; the female elderly have a lower mortality risk than the male elderly. All of them are significant at the level of 1%. Next, the elderly with spouses have a lower mortality risk than the widowed elderly, indicating the protective effect of marriage on health, but it is not significant at the level of 10%. Compared with the control group, those who are educated, living with the offspring and receiving the main source of income from the offspring have a much lower mortality risk. Those who need aids in daily life have a higher mortality risk than those who do not; those with good self-rated health have a lower mortality risk than those with poor self-rated health. These two variables are significant at the level of 1%. Three lifestyle variables—smoke, drink alcohol, and physical exercise—either increase or decrease the mortality risk of the elderly, but they are not significant at the level of 10%.

### Sub Samples: Samples of Different Age Groups

In this part, we categorize the samples into different age groups and analyze the impact of the social old-age insurance and the social medical insurance on them. The results are shown in Table 3.

**Table 3:** The regression results of the Cox model and the frailty Cox model under the samples of different age structures.

**Variable**	**Cox model**				**Frailty Cox model**			
	**Age between 65 and 79**		**Age between 80 and 105**		**Age between 65 and 79**		**Age between 80 and 105**	
	**Model 5**	**Model 6**	**Model 7**	**Model 8**	**Model 9**	**Model 10**	**Model 11**	**Model 12**
The social old-age insurance	−1.31***	−0.02	−1.26***	0.17*	0.70	−1.40*	0.74**	−0.11
The social medical insurance	−2.32***	−1.74***	−1.36***	−0.82***	−0.47	0.47	−3.42***	−0.51*
Residential area		−0.52***		−0.89***		0.64		−1.19***
Gender		−1.49***		−0.89***		−4.49***		−1.89***
Marital status		−0.35***		−0.14**		−0.24		−0.45
Education level		−1.16***		−1.07***		−3.28***		−2.15***
Residential pattern		−0.51***		−0.86***		−1.31*		−1.58***
Source of Income		−0.21**		−1.07***		−0.51		−1.55***
ADL		0.65***		0.32***		3.97***		0.53*
Self-rated health		−1.18***		−0.86***		−1.52**		−0.91***
Smoke		0.13		0.22***		−0.03		0.30
Drink alcohol		0.24**		0.26***		1.88**		0.47
Physical exercise		0.27***		0.26***		−0.74		0.49*
Frailty factors					5.84***	5.39***	4.80***	4.11***

***, **, and * are significant at the levels of 1%, 5%, and 10% respectively.

Under the samples of different age structures, before adding control variables (Model 5 and Model 7) in Cox model 5-8, both the social old-age insurance and the social medical insurance significantly reduce the mortality risk of the elderly. When the control variables are added (Model 6 and Model 8), the social old-age insurance does not have a significant impact on the mortality risk of the elderly aged below 80, but the social medical insurance still reduces the risk. For the elderly aged 80 and above, the result is quite different: the social old-age insurance significantly increases the mortality risk, whereas social medical insurance reduces it.

For all the age groups in the frailty Cox model, before adding control variables (Model 9 and Model 11), the social old-age insurance increases the mortality risk, but the social medical insurance reduces it. The result is significant in the group aged 80 and above and not so significant in the group aged below 80. After controlling other variables, the social old-age insurance significantly reduces the mortality risk of the elderly aged below 80, as shown in Model 10—the mortality risk of those with the insurance is 25% of that of those without (=exp^−1.40^). Meanwhile, the social medical insurance increases the mortality risk of the elderly aged below 80—the mortality risk of those with the insurance is 1.60 (= exp^0.47^) times higher than that of those without, but it is not significant at the level of 10%. For the elderly aged 80 and above, the social old-age insurance reduces the mortality risk—the mortality risk of those with the insurance is 90% of that of those without (=exp^−0.11^), but it is not significant at the level of 10%, as shown in Model 12. The social medical insurance significantly reduces the mortality risk of the elderly aged 80 and above—the mortality risk of those with the insurance is 60% of that of those without (=exp^−0.51^).Meanwhile, the calculation shows that if the frailty factors are not considered, the effect of the social old-age insurance on the mortality risk of the elderly aged below 80 and the elderly aged 80 and above is overestimated by 73 and 29%, respectively, whereas the effect of the social medical insurance is underestimated by 142 and 16%. Control variables are not the focus of this study so there is no discussion here.

From the perspective of model test, the *p*-values of the LR tests are all 0, indicating that Model 10 is better than Model 6 and Model 12 is better than Model 8. In addition, in model 9-12, the frailty factors are significant at the level of 1%, indicating that frailty factors are certainly valuable in the model. Therefore, we take Model 10 and Model 12 as the final interpretation models under the samples of different age structures.

### Sub Samples: Samples of Urban/Rural Areas

In this part, we analyze the samples of urban/rural areas. The regression result of the Cox model and the frailty Cox model are listed simultaneously in Table 4.

**Table 4:** The regression results of the Cox model and the frailty Cox model under the samples of urban/rural areas.

**Variable**	**Cox model**				**Frailty Cox model**			
	**Urban**		**Rural**		**Urban**		**Rural**	
	**Model 13**	**Model 14**	**Model 15**	**Model 16**	**Model 17**	**Model 18**	**Model 19**	**Model 20**
The social old-age insurance	−1.63***	−0.07	−1.27***	0.07	−0.42	−0.24	1.35***	0.26
The social medical insurance	−1.65***	−1.05***	−1.53***	−1.26***	0.23	1.12*	−3.89**	−0.61*
Age group		0.00		0.17***		0.26		0.80***
Gender		−1.38***		−1.13***		−3.74***		−3.24***
Marital status		−0.22***		−0.40***		−0.89		−0.21
Education level		−1.46***		−1.26***		−3.91***		−2.88***
Residential pattern		−0.98***		−0.78***		−1.50***		−1.09***
Source of Income		−0.53***		−0.96***		−0.40		−1.19***
ADL		0.17*		0.37***		0.55		0.65**
Self-rated health		−1.19***		−0.99***		−1.01**		−0.85***
Smoke		0.28***		0.17**		1.86***		−0.10
Drink alcohol		0.63***		0.16**		0.79		−0.37
Physical exercise		−0.22***		0.43***		−1.30***		0.51*
Frailty factors					5.63***	4.86***	4.90***	4.40***

***, **, and * are significant at the levels of 1%, 5%, and 10% respectively.

Table 4 shows that under the samples of urban/rural areas, before adding other control variables (Model 13 and Model 15) in the Cox model 13-16, the social old-age insurance and the social medical insurance significantly reduce the mortality risk of both the urban and rural elderly. When the control variables are added (Model 14 and Model 16), the social old-age insurance has no significant impact on the mortality risk, but the social medical insurance still reduces the risk immensely. In the frailty Cox model 17-20, for the urban elderly, when the control variables are not added (Model 17), neither the social old-age insurance nor the social medical insurance has a significant impact on the mortality risk at the level of 10%. However, when adding control variables (Model 18), the social medical insurance increases the mortality risk of the urban elderly at the level of 10% and the social old-age insurance is still not significant. For the rural elderly, before adding the control variables (Model 19), both the social old-age insurance and the social medical insurance have a significant impact on the mortality risk at the level of 1%, among which the social old-age insurance significantly increases the mortality risk of the rural elderly but the social medical insurance significantly reduces it. When the control variables are added (Model 20), the impact of the social old-age insurance becomes insignificant, whereas the social medical insurance still significantly reduces the risk at the level of 10%. Meanwhile, the calculation shows that if the frailty factors are not considered, the effect of the social old-age insurance on the mortality risk of the urban elderly and the rural elderly is overestimated by 14 and 23%, respectively, whereas the effect of the social medical insurance is underestimated by 271 and 26%.

From the perspective of model test, the *p*-values of the LR tests are all 0, indicating that Model 18 is better than Model 14 and Model 20 is better than Model 16. In addition, in model 17-20, the frailty factors are significant at the level of 1%, indicating that they are valuable in the model. Therefore, we take Model 18 and Model 20 as the final interpretation model under the samples of urban/rural areas. After controlling other variables, as shown in Model 18, the social old-age insurance reduces the mortality risk of the urban elderly—the mortality risk of those with the insurance is 78% of that of those without (=exp^−0.24^), but it is not significant at the level of 10%. Meanwhile, the social medical insurance significantly increases the mortality risk of the urban elderly—the mortality risk of the elderly with the insurance is 3.06 (=exp^1.12^) times higher than that of those without. Model 20 shows that the social old-age insurance increases the mortality risk of the rural elderly—the mortality risk of those with the insurance is 1.30 (= exp^0.26^) times higher than that of those without, but it is not significant at the level of 10%. On the contrary, the social medical insurance significantly reduces the mortality risk of the rural elderly—the mortality risk of those with the insurance is 54% of that of those without (= exp^−0.61^). Control variables are not the focus of this study so there is no discussion here.

### Sub Samples: Samples of Different Regions

In this part, referring to the map of the National Bureau of Statistics of China^2^, we categorize the samples into three regions: the East, the Middle, and the West. The impact of the social old-age insurance and the social medical insurance on the mortality of the elderly is analyzed with the results shown in Table 5.

**Table 5:** The regression results of the Cox model and the frailty Cox model under the samples of different regions.

**Variable**	**Cox model**			**Frailty Cox model**		
	**East**	**Middle**	**West**	**East**	**Middle**	**West**
	**Model 21**	**Model 22**	**Model 23**	**Model 24**	**Model 25**	**Model 26**
The social old-age insurance	−0.37**	0.43***	0.07	−0.20	−1.50	0.67
The social medical insurance	−0.83***	−1.31***	−1.33***	1.43**	−1.75*	−1.60***
Age group	0.22***	0.14	0.20***	0.52	1.88**	0.48
Residential area	−0.73***	−0.83***	−0.69***	−0.97*	−1.68	−1.17***
Gender	−1.21***	−1.06***	−1.13***	−3.61***	−3.15***	−2.78***
Marital status	−0.65***	−0.38***	−0.15**	−1.48***	0.00	0.26
Education level	−1.36***	−1.21***	−1.10***	−4.17***	−2.46**	−2.32***
Residential pattern	−0.75***	−1.14***	−0.66***	−1.06**	−2.40***	−0.49
Source of Income	−1.09***	−0.86***	−0.70***	−1.09**	−1.52**	−0.46
ADL	0.42***	0.35**	0.51***	0.65	2.66***	0.78**
Self-rated health	−1.15***	−0.81***	−0.88***	−1.27***	−0.26	−1.03***
Smoke	0.31***	0.12	0.14	0.50	−0.42	0.29
Drink alcohol	0.43***	0.05	0.16*	1.22**	−0.69	−0.38
Physical exercise	0.24*	0.60***	0.15**	−0.08	0.17	−0.09
Frailty factors				4.57***	5.13***	4.06***

(1) Considering the stability of the results, the samples in the Northeast are not investigated as the number is quite small; (2) For brevity, the regression results before adding control variables are not listed in the table. ***, **, and * are significant at the levels of 1%, 5%, and 10% respectively.

Table 5 shows that in the Cox model, after controlling other variables, the social old-age insurance significantly reduces the mortality risk of the elderly in the East but increases the risk in the Middle, and it has no significant impact on the risk of the elderly in the West. The social medical insurance significantly reduces the mortality risk of the elderly in all regions with no exception. In the frailty Cox model, the social old-age insurance has no significant impact on the mortality risk of the elderly in the East, the Middle and the West at the level of 10%. The social medical insurance significantly increases the mortality risk of the elderly in the East but reduces the mortality risk of the elderly in the Middle and the West.

From the perspective of model test, the *p*-values of the LR tests are < 0.001, indicating that the frailty Cox model is better than the Cox model. Moreover, the frailty factors in model 24-26 are significant at the level of 1%, indicating that they are valuable in the model. Therefore, we take model 24-26 as the final interpretation model under the samples of different regions. Model 24 shows that in the East, the mortality risk of the elderly with the social old-age insurance is 82% (= exp^−0.20^) of that of those without, but it is not significant at the level of 10%. The mortality risk of the elderly with the social medical insurance is 4.18 times higher than that of those without (= exp^1.43^), which is significant at the level of 5%. Model 25 reveals that in the Middle, the mortality risk of the elderly with the social old-age insurance is 22% (= exp^−1.50^) of that of those without, but it is not significant at the level of 10%. The mortality risk of the elderly with the social medical insurance is 17% (= exp^−1.75^) of that of those without, and it is significant at the level of 10%. Model 26 reveals that in the West, the mortality risk of the elderly with the social old-age insurance is 1.95 times higher than that of those without (= exp^0.67^), but it is not significant at the level of 10%. The mortality risk of the elderly with the social medical insurance is 20% (= exp^−1.60^) of that of those without, and it is significant at the level of 1%. Meanwhile, the calculation shows that if the frailty factors are not considered (Model 21-23), the effect of the social old-age insurance on the mortality risk of the elderly in the East, Middle and West is underestimated by 13%, overestimated by 131% and underestimated by 88%, respectively, whereas the effect of the social medical insurance is underestimated by 374%, overestimated by 10% and overestimated by 6%, respectively. Other variables are not the focus of this study so there is no discussion here.

## Discussion and Conclusion

As mentioned above, the main purpose of this paper is to investigate the heterogeneity of the social old-age insurance and the medical insurance on the mortality risk of the elderly in different ages and different regions under the background of the increasing aging in China.

Inspired by the previous studies, this paper uses the four-period follow-up data from CLHLS 2008-2017/2018 in the Cox proportional risk model and Cox proportional risk model with frailty factors, and investigates the impact of the social old-age insurance and the social medical insurance on the mortality risk of the elderly from four perspectives: the overall samples, samples of different age structures, samples of urban/rural areas and samples of different regions. The comparative analysis of the results show that it is necessary to consider frailty factors in the studies of the influential factors of the mortality risk. When other factors are controlled, including frailty factors, the social old-age insurance and the social medical insurance do not have a significant impact on the mortality risk of the elderly in general. Under the samples of different age structures, the social old-age insurance significantly reduces the mortality risk of the elderly aged below 80, but it has no significant effect on the elderly aged 80 and above; the social medical insurance has no significant impact on the mortality risk of the elderly aged below 80, but significantly reduces the mortality risk of the elderly aged 80 and above. Under the samples of urban/rural areas, the social old-age insurance has no significant impact on the mortality risk of both the urban and rural elderly; the social medical insurance significantly increases the mortality risk of the urban elderly but significantly reduces the mortality risk of the rural elderly. Under the samples of different regions, the social old-age insurance has no significant impact on the mortality risk of the elderly in the East, the Middle and the West; the social medical insurance significantly increases the mortality risk of the elderly in the East but significantly reduces the mortality risk of the elderly in the Middle and the West.

Under the overall samples, neither the social old-age insurance nor the social medical insurance has a significant impact on the mortality risk of the elderly. China has a vast territory, meaning that different regions have different economic development levels, different resources of insurances, different scales of social security, and adverse selections of insurance. The insured population are more likely to be those in poor physical condition or those with higher mortality risk (34, 35). All of these factors are so sophisticated that they offset the significance of the two insurances on the mortality risk of the elderly.

Under the samples of different age structures, the social old-age insurance significantly reduces the mortality risk of the elderly aged below 80. They have a longer period to enjoy various health benefits brought by a steady stream of pensions to preserve their health and go traveling. For the elderly aged 80 and above, the declining function of organs limits their health benefits, such as hiking and traveling. At this stage, they are no longer proud of the amount of pension they get, but calculate the years of pension they can get for the rest of their life. Therefore, the social old-age insurance has no significant impact on their mortality risk. On the other hand, the social medical insurance has no significant impact on the mortality risk of the elderly aged below 80. The possible reason is that the elderly at this age are relatively healthy and do not need the insurance. Therefore, the social medical insurance has little impact on their mortality risk. However, the social medical insurance is of great importance for the elderly aged 80 and above. Due to the declining function of physical health, they suffer from geriatric diseases, and the medical expenses or nursing expenses are increasing. The support of the social medical insurance will significantly reduce their mortality risk.

Under the samples of urban/rural areas and the samples of different regions, the impact of the social old-age insurance and the social medical insurance on the mortality risk of the elderly is heterogeneous. The purpose of the social old-age insurance is to ensure the basic living needs of the elderly and provide them with a stable and reliable source of income. The elderly can spend their pension as they want. For the urban elderly and the elderly in the East, their pension is mainly used in daily expenses or medical service expenses (36). Some elderly with high pension also use it for health preservation or traveling. Therefore, the impact of the social old-age insurance is not significant on the mortality risk of the urban elderly and the elderly in the East. There could be another explanation that the urban elderly and the elderly in the East are mostly people with better economic conditions. They have accumulated a lot of assets or wealth when they were young. Their consumption in the retirement stage mainly depends on the early savings, and the pension is insignificant compared to that. Therefore, the social old-age insurance has little impact on their mortality risk. For the majority of the rural elderly and the elderly in the Middle and the West, the amount of pension is generally small, which is not enough to meet their daily expenses, let alone health preservation or traveling. Therefore, the impact on the mortality risk of the rural elderly and the elderly in the Middle and the West is also insignificant. Different from the social old-age insurance, the social medical insurance is not obtained from withdrawals, but from the reimbursement of medical expenditure, which is mainly used for medical treatment such as illness, severe disease and recovery treatment. The social medical insurance in China develops from the urban areas to the rural areas. As the medical resources are highly concentrated in the urban areas and economically developed areas, and the security level is higher than that in rural and economically underdeveloped areas, we assume that the mortality risk of the urban elderly or the elderly in the East with the social medical insurance should be lower than that of the urban elderly without the insurance, because they enjoy good medical skills, high-end medical devices and high reimbursement level. However, the statistical results show that the mortality risk of the former is significantly higher than that of the latter. The possible reason is the malfunction of price controls, which leads to high prices of medicines, excessive medical treatment, rapid rise of medical expenses (37, 38) and excessive medical treatment (such as excessive physical examination, excessive use of drugs, etc.) induced by the doctors (suppliers) (39, 40). Another possible reason is the excessive demand of the patient, that is, the insured elderly tends to have excessive demand of treatment due to low medical costs (41, 42). On the other hand, the social medical insurance greatly reduces the mortality risk of the rural elderly and the elderly in the Middle and the West. The possible reason is that the social medical insurance develops late in these regions, and the elderly have few notions of it (they do not go to the hospital unless they are very ill). Now the social medical insurance alleviates their economic pressure to a great extent, reduces their spiritual burden, and eliminates the idea of “no need to go to the hospital though we are sick” (7). In addition, the improvement of medical technology, the development of medical equipment and the strengthening of medicinal efficacy largely enhance the impact of the social medical insurance on reducing the mortality risk of the rural elderly and the elderly in the Middle and the West.

As mentioned above, the whole population in China is covered by the Basic Old-age Insurance for Urban and Rural Residents in 2014 and then by the Basic Medical Insurance for Urban and Rural Residents by the end of 2020. We can see the great achievements in social security. However, this study shows that the health benefits of the social old-age insurance and the social medical insurance vary in age structures, urban/rural areas and regions, meaning that the Chinese government and responsible departments should guarantee the insurance among the targeted elderly based on their attributes. The share of medical reimbursement should be increased, especially the recovery and nursing expenses. What is more, it is necessary to give a full consideration in the investment and the resource allocation of social security to balance the supply and demand of medical resources; it is urgent to reduce the competition among doctors in hospitals in economically developed areas and big cities to control the excessive medical induction; it is mandatory to carry out the publicity for the elderly in the East and urban areas to reduce their excessive medical demand, so as to relieve the pressure of medical treatment. For the rural areas and the Middle and the West, it is necessary to reallocate the medical resources, increase medical facilities, enlarge medical service personnel, and gradually improve the pension level. In addition, it is a must to continuously improve the service and security quality of the social insurances and the social security system, then finally achieve the goal of healthy aging and health equity.

In the end, though this empirical research shows that the mortality risk of the urban elderly and the elderly in the East with the social medical insurance is higher than that of those without, it does not mean that the social medical insurance implemented in China has failed. The results of this paper only indicate a possibility for that. The possible reasons are the excessive medical treatment and demand mentioned above, or the adverse selections of insurance. For the urban elderly and the elderly in the East, further studies shall focus on: How does the excessive medical treatment and demand offset the health benefits brought by the social medical insurance in reducing the mortality risk of the elderly? How do the medical level, medical facilities, doctors and medical needs influence on the mortality risk of the elderly? What is the functional direction and degree of these factors? What is the relation between these factors? How do we measure the adverse selection of insurance and its impact on the mortality risk? All of these questions are waiting for further observation and answers.

## Data Availability Statement

The raw data supporting the conclusions of this article will be made available by the authors, without undue reservation.

## Author Contributions

BG is responsible for data collection and policy analysis. CL is responsible for data analysis. WW is responsible for providing overall ideas. All authors contributed to the article and approved the submitted version.

## Funding

This work was supported by the Chinese Longitudinal Healthy Longevity Study (CLHLS) datasets analyzed and the research reported in this paper are jointly supported by the National Key R&D Program of China (2018YFC2000400), National Natural Sciences Foundation of China (71490732), the U.S. National Institute of Aging of National Institute of Health (P01AG031719), the Natural Science Foundation of Guangxi Province of China (AD20159052), Guangxi Philosophy and Social Science Planning Research Project (20FRK001), and University-level Scientific Research Project of Guangxi University for Nationalities (2020MDSKZD02).

## Conflict of Interest

The authors declare that the research was conducted in the absence of any commercial or financial relationships that could be construed as a potential conflict of interest.

## Publisher's Note

All claims expressed in this article are solely those of the authors and do not necessarily represent those of their affiliated organizations, or those of the publisher, the editors and the reviewers. Any product that may be evaluated in this article, or claim that may be made by its manufacturer, is not guaranteed or endorsed by the publisher.

## Footnotes

^1^Website of the Pecking University Research Open Data: https://opendata.pku.edu.cn/dataverse/CHADS.

^2^Website of the National Bureau of Statistics: http://www.stats.gov.cn/tjsj/zxfb/201405/t20140527_558611.html.
